# The Fluorescent Detection of Glucose and Lactic Acid Based on Fluorescent Iron Nanoclusters

**DOI:** 10.3390/s24113447

**Published:** 2024-05-27

**Authors:** Jing Ge, Wenlu Mao, Xinyi Wang, Muqi Zhang, Siyu Liu

**Affiliations:** College of Life and Health Sciences, Northeastern University, Shenyang 110004, China; yywq1912@163.com (J.G.); maowenlu0825@163.com (W.M.); wangxinyi20030901@163.com (X.W.); 15802412551@163.com (M.Z.)

**Keywords:** iron nanoclusters, fluorescence, peroxide-like activity, glucose, lactic acid

## Abstract

In this paper, a novel fluorescent detection method for glucose and lactic acid was developed based on fluorescent iron nanoclusters (Fe NCs). The Fe NCs prepared using hemin as the main raw material exhibited excellent water solubility, bright red fluorescence, and super sensitive response to hydrogen peroxide (H_2_O_2_). This paper demonstrates that Fe NCs exhibit excellent peroxide-like activity, catalyzing H_2_O_2_ to produce hydroxyl radicals (^•^OH) that can quench the red fluorescence of Fe NCs. In this paper, a new type of glucose sensor was established by combining Fe NCs with glucose oxidase (GluOx). With the increase in glucose content, the fluorescence of Fe NCs decreases correspondingly, and the glucose content can be detected in the scope of 0–200 μmol·L^−1^ (μM). Similarly, the lactic acid sensor can also be established by combining Fe NCs with lactate oxidase (LacOx). With the increase in lactic acid concentration, the fluorescence of Fe NCs decreases correspondingly, and the lactic acid content can be detected in the range of 0–100 μM. Furthermore, Fe NCs were used in the preparation of gel test strip, which can be used to detect H_2_O_2_, glucose and lactic acid successfully by the changes of fluorescent intensity.

## 1. Introduction

Glucose, as a monosaccharide, is the primary energy source for cellular metabolism [1]. Glucose level fluctuations in our body fluids are usually associated with numerous diseases, including hypoglycemia, diabetes, and so on [2,3,4,5]. For example, glucose serum content in healthy people and diabetic patients are usually in the concentration range of 3–8 mmol·L^−1^ (mM) and 2–40 mM [6]. By accurately measuring glucose levels in diabetic patients, their diabetes can be effectively monitored and treated [7].

Lactic acid is a byproduct of anaerobic glycolysis in tissues, and its content can be viewed as an indicator of the oxygenation level of tissues [8]. The fluctuations of lactic acid levels in the body are frequently linked to various human disorders, such as acidosis [9]. Recently, lactic acid concentrations in human blood were recommended as an indicator for evaluating the therapeutic and prognostic effects in COVID-19 [10]. Besides, lactic acid can be generated by bacterial fermentation in food products [11]. Therefore, it is crucial to monitor glucose and lactic acid levels quickly and accurately in biological fluids such as blood, urine, tears, etc., and foodstuffs.

Until now, various technologies have been utilized for detecting glucose and lactic acid content, such as capillary electrophoresis, gas chromatography, high-performance liquid chromatography, etc. [12,13,14]. Electrochemical assays have been widely used for detecting glucose and lactic acid due to their convenient operation and fast response [15,16,17,18]. For example, Liao et al. developed an electrochemical sensor for detecting glucose by depositing Pt nanoparticles on a SiC@C nanowire and immobilizing GluOx [15]. Hsu et al. prepared a enzymatic glucose fiber sensor that could detect glucose concentration in the range of 10–550 mg/L on the basis of amount of phase variation [16]. Abrar and co-workers developed an amperometric lactate biosensor by modifying silver electrode with lactate oxidase, which shows the linear response to lactate concentration of 1–25 mM [17]. However, these electrodes need to be cleaned repeatedly after the detection in biological fluid samples, and are not suitable for the preparation of disposable sensors [18]. Fluorescent sensors have attracted widespread attention in recent years due to their features of high detection sensitivity, good selectivity, simple operation, low cost, fast response speed, and the absence of complex pre-processing requirements. Based on the detection principle, glucose or lactic acid sensors can be mainly categorized mainly into two types. Enzymatic sensors commonly utilize specific enzymes such as GluOx and LacOx to catalyze the oxidation of glucose or lactic acid. These sensors can measure downstream products such as H_2_O_2_ resulting from glucose or lactic acid [19,20,21,22,23,24]. For example, Zhang et al. developed a visual platform comprising MnO_2_ nanosheets, GluOx, and rhodamine B for detecting glucose ranged from 0.25 to 20 μM based on the emission wavelength shift of rhodamine B [25]. Su’s team constructed a detection system consisting of Cu-doped nanozyme (CuAA) and Mg/N-doped carbon dots (Mg-N-CQDs). CuAA can catalyze the conversion of non-fluorescent o-phenylenediamine (OPD) to fluorescent 2,3-diaminophenazine (DAP) in the presence of H_2_O_2_, which is generated following the oxidation of glucose catalyzed by GluOx. The fluorescence around 444 nm of Mg-N-CQDs was quenched due to the production of DAP through an inner-filter effect. The glucose concentration can be detected in the range of 2–400 μM based on the fluorescence ratio I_558_/I_444_ of CuAA, Mg-N-CQDs, and the GluOx system [26]. Zhao et al. provide a mixed system based on the formation of silver nanoclusters for the programmable determination of a series of substrates, including glucose, uric acid, lactic acid, etc. [27]. Non-enzymatic biosensors, which do not rely on enzymes, can directly detect glucose or lactic acid through fluorescent changes [28]. Li et al. developed a hydrogel sensor functionalized with a fluorescein derivative and CdTe QDs/3-APBA, which can detect glucose concentrations in the range of 0–20 mM [29]. Kshtriya et al. developed acyl-thiourea-based assembled fluorescent fibers, whose fluorescence color can be disrupted in the presence of Cu^2+^ ions and further recovered by the continuous addition of lactic acid [30].

Fluorescent iron nanoclusters (Fe NCs), as an emerging fluorescent nanomaterial, display ultra-small size, well water solubility, and excellent antibacterial activity. In previous studies, we reported red-emitting Fe NCs with hemin as the main raw material, which displayed a broad spectrum of activity against Gram-positive bacteria [31]. Herein, we demonstrated the more sensitive response of fluorescent Fe NCs to external H_2_O_2_ concentration than potassium dichromate, and further confirmed its peroxide-like activity. Based on the fluorescence quenching phenomenon induced by H_2_O_2_, we further established a detection system for glucose and lactic acid by combining Fe NCs with GluOx or LacOx.

## 2. Experimental Detail

### 2.1. Synthesis of Fe NCs

The Fe NCs were synthesized as the following process [31]. An amount of 2 mg of hemin was dissolved in 17 mL of NaOH solution (3.5 mM) and then intensively mixed with 2 mL of solution containing 52 mg of L-histidine (His) and 10 mg of sodium borohydride at 25 °C. The pH value of the mixed solution was 11.2. After 10 h of reaction time, the mixture was purified using ultrafiltration centrifugation (1000 Da cutoff) and lyophilization.

### 2.2. Detection of H_2_O_2_

For the detection of H_2_O_2_, 20 μL of Fe NCs solution (1.0 mg·mL^−1^) and 20 μL of H_2_O_2_ solutions with different concentrations were mixed with 100 μL of Na_2_HPO_4_–NaH_2_PO_4_ buffered solution (PBS) (pH 7.4, 200 mM) and further diluted to 200 μL with deionized water while being continuously vibrated for 10 min. All the operations were performed at 25 °C.

### 2.3. Detection of Glucose

For the analysis of glucose concentration, 20 μL of Fe NCs solution (1.0 mg·mL^−1^), various volumes of GluOx solution, and glucose solutions were mixed with 100 μL of PBS (pH 7.4, 200 mM) and further diluted to 200 μL with deionized water. The mixture was continuously vibrated for 60 min at 25 °C.

### 2.4. Detection of Lactic Acid

For the analysis of lactic acid concentration, 20 μL of Fe NCs solution (1.0 mg·mL^−1^), various volumes of LacOx solution, and lactic acid solutions were mixed with 100 μL of PBS (pH 7.4, 200 mM) and further diluted to 200 μL with deionized water. The mixture was continuously vibrated for 60 min at 25 °C.

### 2.5. The Preparation of Agarose Gel Based on Fe NCs

In total, 1.0 g of agarose was dissolved in 40 mL of boiled deionized water. An amount of 1.5 mL of hot agarose solution was added into 6-well plate. As the agarose solution was cooled to a temperature of 40 °C, 400 µL of Fe NCs solution (1.0 mg·mL^−1^) and 100 µL of deionized water were injected into the agarose solution. After the mixture was cooled down to room temperature, the agarose gel disc in a 6-well plate with a radius of 1.425 cm was obtained. The prepared agarose gel disc was wrapped in a plastic wrap and preserved at 4 °C. The prepared agarose gel discs containing different amounts of Fe NCs were placed on the plastic plate, and their color changes were observed using a dark-box ultraviolet analyzer upon 365 nm irradiation.

### 2.6. The Preparation of Agarose Gel for Glucose Based on Fe NCs and GluOx

In total, 1.0 g of agarose was dissolved in 40 mL of boiled deionized water. An amount of 1.5 mL of hot agarose solution was added into 6-well plate. As the agarose solution was cooled to a temperature of 40 °C, 400 µL of Fe NCs solution (1.0 mg·mL^−1^) and 100 µL of GluOx solution (0.5 mg·mL^−1^) were injected into the agarose solution. After the mixture was cooled down to room temperature, the agarose gel disc in a 6-well plate was obtained. The prepared agarose gel disc was wrapped in a plastic wrap and preserved at 4 °C. The series of agarose gel discs containing Fe NCs and GluOx were placed on the plastic plate, and 200 µL of different concentrations of glucose solution (0–400 µM) were injected into these agarose gel discs, respectively. After one hour of incubation, the color changes of the agarose gel discs were observed using a dark-box ultraviolet analyzer upon 365 nm irradiation.

### 2.7. The Preparation of Agarose Gel for Lactic Acid Based on Fe NCs and LacOx

In total, 1.0 g of agarose was dissolved in 40 mL of boiled deionized water. An amount of 1.5 mL of hot agarose solution was added into a 6-well plate. As the agarose solution was cooled to a temperature of 40 °C, 400 µL of Fe NCs solution (1.0 mg·mL^−1^) and 100 µL of LacOx solution (0.10 mg·mL^−1^) were injected into the agarose solution. After the mixture was cooled down to room temperature, the agarose gel disc in a 6-well plate was obtained. The prepared agarose gel disc was wrapped in plastic wrap and preserved at 4 °C. The series of agarose gel discs containing Fe NCs and LacOx were placed on the plastic plate, and 200 µL of different concentrations of lactic acid solution (0–400 µM) were injected into these agarose gel discs, respectively. After one hour of incubation, the color changes of the agarose gel discs were observed using a dark-box ultraviolet analyzer upon 365 nm irradiation.

### 2.8. The Determination of Glucose and Lactic Acid in Fetal Calf Serum Samples and Milk Samples

The commercial fetal calf serum was further diluted 20 times using PBS (pH 7.4, 100 mM), and spiked with different amounts of glucose or lactic acid. For the analysis of glucose in serum samples, 20 μL of Fe NCs solution (1.0 mg·mL^−1^) and 20 µL of GluOx solution (100 µg·mL^−1^) were mixed with 100 μL of the above serum samples and further diluted to 200 μL with deionized water. The mixture was continuously vibrated for 60 min at 25 °C. Similarly, for the analysis of lactic acid in serum samples, 20 μL of Fe NCs solution (1.0 mg·mL^−1^) and 20 µL of LacOx solution (20 µg·mL^−1^) were mixed with 100 μL of the above serum samples and further diluted to 200 μL with deionized water. The mixture was continuously vibrated for 60 min at 25 °C.

The commercial milk purchased at a local supermarket was further diluted 20 times using PBS (pH 7.4, 100 mM) and spiked with different amounts of glucose or lactic acid. For the analysis of glucose in milk samples, 20 μL of Fe NCs solution (1.0 mg·mL^−1^) and 20 µL of GluOx solution (100 µg·mL^−1^) were mixed with 100 μL of the above milk samples and further diluted to 200 μL with deionized water. The mixture was continuously vibrated for 60 min at 25 °C. Similarly, for the analysis of lactic acid in milk samples, 20 μL of Fe NCs solution (1.0 mg·mL^−1^) and 20 µL of LacOx solution (20 µg·mL^−1^) were mixed with 100 μL of the above milk samples and further diluted to 200 μL with deionized water. The mixture was continuously vibrated for 60 min at 25 °C.

## 3. Results and Discussion

**Scheme 1:**
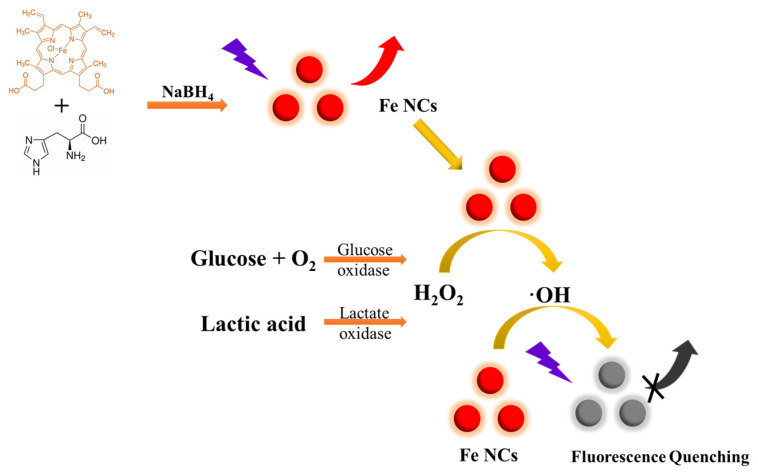
The illustrations of the synthesis of Fe NCs and the detection for glucose and lactic acid.

**Figure 1:**
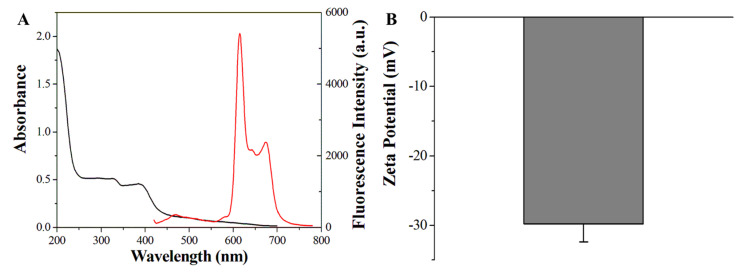
(**A**) The fluorescence emission spectra (red line) and UV-Vis adsorption spectra (black line) of Fe NCs. (**B**) Zeta potential measurement of Fe NCs solutions.

**Figure 2:**
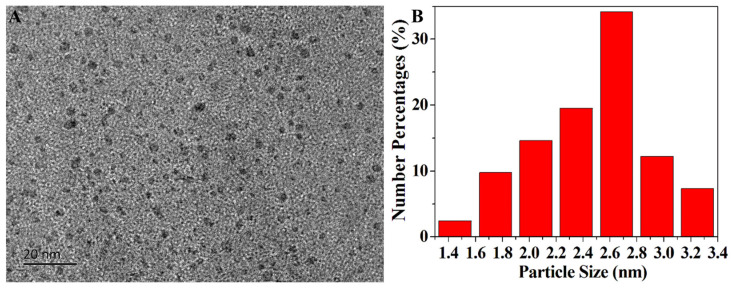
(**A**) TEM images of Fe NCs. (**B**) The particle size statistics histogram of Fe NCs.

As shown in the Scheme 1, the Fe NCs was synthesized utilizing hemin as raw material, His as an extractant, and sodium borohydride as a reducing agent [32,33]. The prepared Fe NCs core was modified by hemin and His [31]. As shown in Figure 1A, the Fe NCs displayed three main fluorescence emission peak at 616 nm, 645 nm, and 675 nm, respectively, with the excitation wavelength of 400 nm. There was an evident absorption band around 395 nm in the UV-Vis absorption spectra. As shown in Figure 1B, the zeta potential measurements of Fe NCs solution was −29.8 mV, indicating the negative charges on the Fe NCs surface and colloidal stability in the aqueous solution. The appearance of Fe NCs was characterized using transmission electron microscopy (TEM). As shown in Figure 2, the prepared Fe NCs exhibited typical spherical shape with well dispersity. The corresponding particle size statistics histogram manifested that the particle size of Fe NCs ranged from 1.48 nm to 3.36 nm, with an average size of 2.49 nm. The above results showed that we successfully prepared fluorescent Fe NCs, which is consistent with previous report [31].

**Figure 3:**
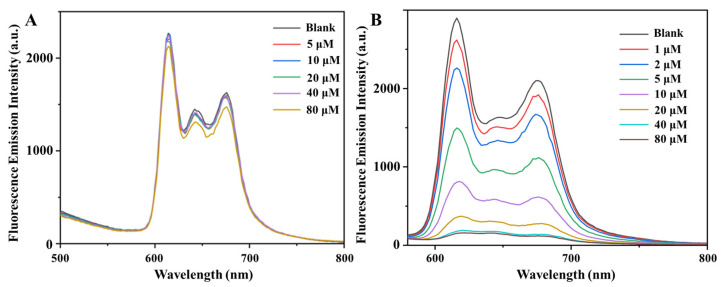
The fluorescence emission spectra of Fe NCs incubated with different concentrations of (**A**) potassium dichromate or (**B**) hydrogen peroxide for 10 min.

**Figure 4:**
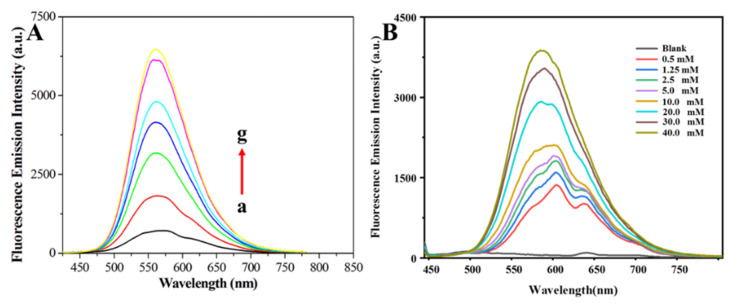
(**A**) The fluorescence emission spectra of OPD-H_2_O_2_-Fe NCs system. OPD: 40 mM, H_2_O_2_: 10 μM, Fe NCs: 1 μg·mL^−1^ (a), 2.5 μg·mL^−1^ (b), 5 μg·mL^−1^ (c), 7.5 μg·mL^−1^ (d), 10 μg·mL^−1^ (e), 20 μg·mL^−1^ (f), and 40 μg·mL^−1^ (g). Incubation time was 30 min. (**B**) The fluorescence emission spectra of OPD-H_2_O_2_-Fe NCs system. H_2_O_2_: 10 μM, Fe NCs: 7.5 μg·mL^−1^, OPD: 0 mM, 0.5 mM, 1.25 mM, 2.5 mM, 5.0 mM, 10.0 mM, 20.0 mM, 30.0 mM, and 40.0 mM. Incubation time was 30 min. All the operations were performed at 25 °C.

As shown in Figure 3, we investigated the influence of different concentrations of potassium dichromate and H_2_O_2_ on the fluorescence of Fe NCs. In the concentration range of 0–80 μM, potassium dichromate had little influence on the fluorescence of Fe NCs, while H_2_O_2_ significantly quenched its fluorescence. Considering that the standard electrode potential φ^θ^(Cr_2_O_7_^2−^/Cr^3+^) = 1.33 eV and φ^θ^(H_2_O_2_/H_2_O) = 1.77 eV, we speculate that some stronger oxidants, such as ^•^OH(φ^θ^(^•^OH/H_2_O) = 2.87 eV) may be generated in the presence of Fe NCs and H_2_O_2_. In order to test this conjecture, we introduced Fe NCs as a catalyst into the reaction system consisting of o-phenylenediamine (OPD) and H_2_O_2_. As shown in the Scheme 1, if H_2_O_2_ can be converted into the highly reactive radical ^•^OH, OPD will be quickly oxidized by ^•^OH to generate its oxidized product OPDox, which displays bright yellow fluorescence around 565 nm [34,35]. As shown in Figure 4A, we explored the influence of various concentrations of Fe NCs on the fluorescence of the OPD-H_2_O_2_ system. It could be observed that with the increase in Fe NCs concentrations from 1 to 40 μg·mL^−1^, the fluorescence emission peak around 565 nm attributed to OPDox enhanced gradually. Similarly, Figure 4B presented the fluorescence emission spectra of the Fe NCs-H_2_O_2_ system incubated with various OPD concentrations, as the OPD concentrations increased from 0 to 40 mM, the fluorescence emission peak around 565 nm attributed to OPDox also increased accordingly. The above results indicate that Fe NCs could serve as a peroxidase, converting H_2_O_2_ into ^•^OH.

**Figure 5:**
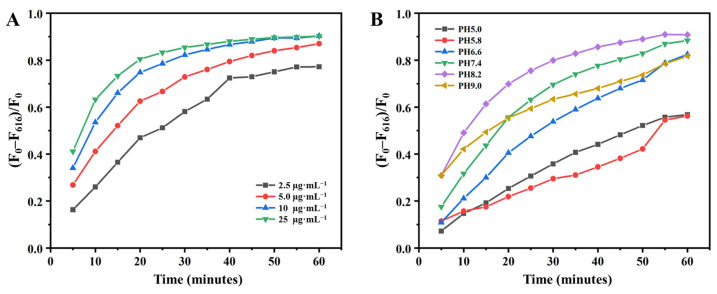
(**A**) The fluorescence intensity changes of Fe NCs solution incubated with 100 µM glucose and different concentrations of GluOx (2.5, 5.0, 10, 25 µg·mL^−1^) at different reaction times (pH 7.4, 100 mM PBS). (**B**) The fluorescence intensity changes of Fe NCs solution incubated with 100 µM glucose and 10 µg·mL^−1^ of GluOx in different pH environments at different reaction time (100 mM PBS). All the operations were performed at 25 °C.

As shown in Figure 3B and Scheme 1, the Fe NCs can not only be used as a fluorescent probe for detecting H_2_O_2_, but also serve as a peroxidase to convert H_2_O_2_ into ^•^OH. It is well-known that GluOx can serve as a catalyst used for the conversion of glucose to gluconic acid and H_2_O_2_. Herein, we propose an assay system composed of Fe NCs and GluOx for the application in glucose detection. Firstly, we investigated the fluorescence intensity changes of Fe NCs solution incubated with 100 µM glucose and different concentrations of GluOx (2.5, 5.0, 10, 25 µg·mL^−1^) along with the extension of incubation time. F_0_ represents the initial fluorescence intensity at 616 nm of Fe NCs, while F_616_ indicates the fluorescence intensity at 616 nm of Fe NCs after incubation with glucose and GluOx at the scheduled time. As shown in Figure 5A, with the increase in GluOx concentration from 2.5 to 25 µg·mL^−1^, the proportion of fluorescence quenching (F_0_–F_616_)/F_0_ gradually increased at the same time point. Along with extending incubation time to 60 min, the fluorescence intensity changes in Fe NCs became stable. Therefore, 60 min of incubation time was adopted in the following experiments. The fluorescence quenching induced by glucose was on the basis of the combined action of GluOx and Fe NCs with peroxide activity. It is well known that pH environments have significant effects on the activity of enzymes [36,37]. Herein, we further investigated the changes in fluorescence intensity of Fe NCs solution incubated with 100 µM glucose and 10 µg·mL^−1^ of GluOx in different pH environments at various reaction times. Figure 5B demonstrates that the proportion of fluorescence quenching (F_0_–F_616_)/F_0_ in a slightly acidic environment (pH 5.0 and pH 5.8) was lower than in neutral and alkaline environments (pH 6.6, pH 7.4, pH 8.2, pH 9.0). Based on the above experimental results, 10 µg·mL^−1^ of GluOx and a pH 7.4 buffer environment were selected to assay glucose in the following procedure.

**Figure 6:**
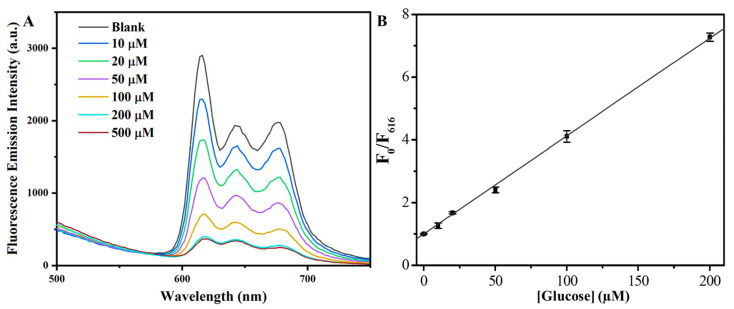
(**A**) The fluorescence emission spectra of Fe NCs solution incubated with 10 µg·mL^−1^ of GluOx, and different concentrations of glucose for 60 min (pH 7.4, 100 mM PBS). (**B**) The linear relationship between F_0_/F_616_ and glucose concentrations. All the operations were performed at 25 °C.

The fluorescent changes in Fe NCs and the GluOx system’s response to various glucose concentrations were illustrated in Figure 6. It can be observed that with the rising in glucose concentration, the fluorescence emission peak around 616 nm decreases gradually. As shown in Figure 6B, the relationship between the fluorescence intensity at 616 nm and glucose concentrations in the range of 0–200 μM can be well-described in the following equation:

F_0_/F_616_ = 0.0312 [Glucose] + 1.0060 (1)

**Figure 7:**
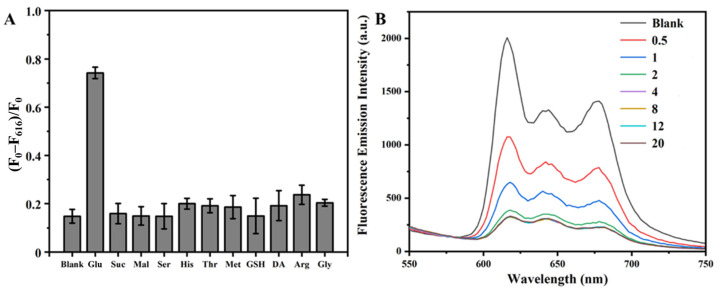
(**A**) The fluorescence intensity changes of Fe NCs and the GluOx system incubated with 100 μM different additional biomolecules, respectively, for 60 min. Fe NCs: 100 μg·mL^−1^, GluOx: 10 µg·mL^−1^, pH environments: 7.4. (**B**) The fluorescence emission spectra of Fe NCs solution incubated with 100 μM of lactic acid, and different concentrations of LacOx (0, 0.5, 1, 2, 4, 8, 12, 20 μg·mL^−1^) for 60 min (pH 7.4, 100 mM PBS). All the operations were performed at 25 °C.

F_0_ is the initial fluorescence intensity at 616 nm of Fe NCs, while F_616_ indicates the fluorescence intensity at 616 nm of Fe NCs after incubation with 10 µg·mL^−1^ of GluOx and various concentrations of glucose. The detection limit for glucose was 1.6 μM, and the fitting coefficient was 0.999. In order to test the selectivity of this method for glucose detection, we investigated the fluorescent response of the Fe NCs–GluOx system to a series of biomolecules (100 μM), including glucose (Glu), sucrose (Suc), maltose (Mal), serine (Ser), His, threonine (Thr), methionine (Met), glutathione (GSH), dopamine (DA), arginine (Arg), and glycine (Gly). Figure 7A shows that only glucose could significantly quench the fluorescence of the detection system, demonstrating the well-selectivity of this method for glucose.

**Figure 8:**
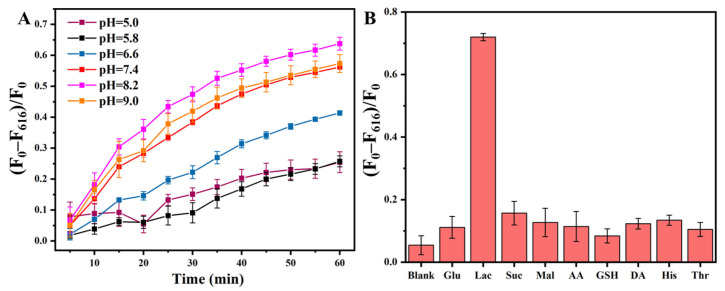
(**A**) The fluorescence intensity changes of Fe NCs solution incubated with 50 µM lactic acid and 2 µg·mL^−1^ of LacOx in different pH environments at different reaction times (100 mM PBS). (**B**) The fluorescence intensity changes of Fe NCs and the LacOx system incubated with 100 μM different additional biomolecules, respectively, for 60 min. Fe NCs: 100 μg·mL^−1^, LacOx: 2 µg·mL^−1^, pH environments: 7.4.

As shown in the Scheme 1, LacOx can serve as a catalyst used for the conversion of lactic acid to H_2_O_2_ and pyruvic acid [27]. Herein, we further investigated the feasibility of an assay system composed of Fe NCs and LacOx for the detection of lactic acid. Figure 7B shows the fluorescence emission spectra of Fe NCs solution incubated with 100 μM of lactic acid and various concentrations of LacOx ranged from 0 to 20 μg·mL^−1^ for 60 min. It can be observed that the fluorescence intensity of Fe NCs solution weakened correspondingly as the concentration of LaxOx increased from 0 to 4 μg·mL^−1^. Thus, the fluorescence quenching induced by lactic acid was on the basis of the combined action of LaxOx and Fe NCs with peroxide activity. Herein, we investigated the changes in fluorescence intensity of Fe NCs solution when incubated with lactic acid and 2 µg·mL^−1^ of LacOx in various pH environments at different reaction times. Figure 8A shows that the proportion of fluorescence quenching (F_0_–F_616_)/F_0_ in slightly acidic environments (pH 5.0, pH 5.8) varies slightly with the reaction time, and increases noticeably in neutral and alkaline environments (pH 6.6, pH 7.4, pH 8.2, pH 9.0). In the subsequent experiments, 2 µg·mL^−1^ of LacOx and a pH 7.4 buffer environment were used to assay lactic acid. The fluorescent response of the Fe NCs–LacOx system to a series of biomolecules (100 μM), including Glu, lactic acid (Lac), Suc, Mal, ascorbic acid (AA), GSH, DA, His, and Thr, is provided in Figure 8B. It was found that only lactic acid could significantly quench the fluorescence of the detection system composed of Fe NCs and LacOx, demonstrating the high selectivity of this method for LacOx.

**Figure 9:**
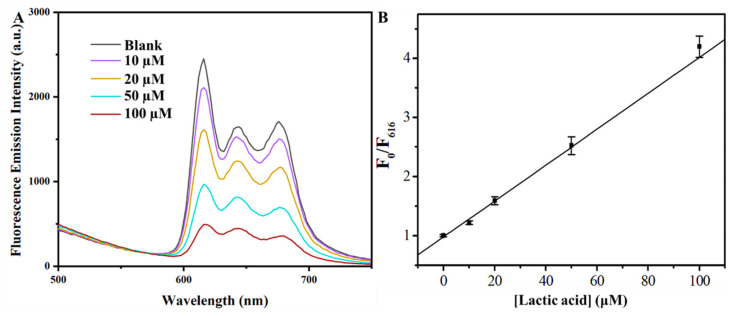
(**A**) The fluorescence emission spectra of Fe NCs solution incubated with 2 µg·mL^−1^ of LacOx, and different concentrations of lactic acid for 60 min (pH 7.4, 100 mM PBS). (**B**) The linear relationship between F_0_/F_616_ and lactic acid concentrations. All the operations were performed at 25 °C.

The fluorescence emission spectra of Fe NCs and the LacOx system incubated with various concentrations of lactic acid are provided in Figure 9. Along with the increase in lactic acid concentration, the fluorescence emission peak around 616 nm decreased gradually. As shown in Figure 9B, the relationship between the fluorescence intensity at 616 nm and lactic acid concentrations in the range of 0–100 μM could be well-described by the following equation:

F_0_/F_616_ = 0.0304 [Lactic acid] + 0.9781 (2)

F_0_ is the initial fluorescence intensity at 616 nm of Fe NCs, and F_616_ is the fluorescence intensity at 616 nm of Fe NCs incubated with 2 µg·mL^−1^ of LacOx and different concentrations of lactic acid. The detection limit for lactic acid was 1.5 μM, and the fit coefficient was 0.993. The above results demonstrate that the Fe NCs–GluOx system and the Fe NCs–LacOx system can be successfully used for the selective detection of glucose and lactic acid.

**Figure 10:**
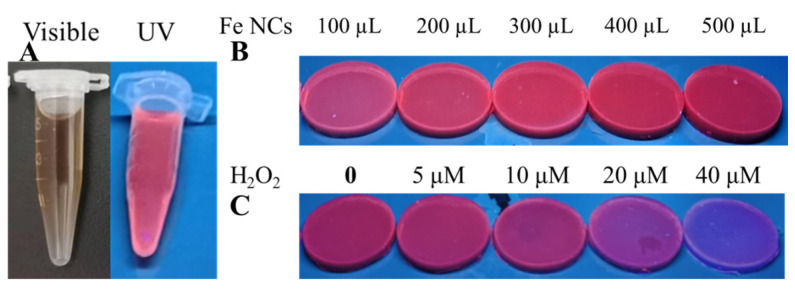
(**A**) Photo of Fe NCs solution under visible light and UV light. (**B**) Photo of agarose gel discs prepared with various volumes of Fe NCs solution (1 mg·mL^−1^) under UV light. (**C**) Photo of agarose gel discs based on Fe NCs immersed in H_2_O_2_ solution with various concentrations for 30 min under UV light.

**Figure 11:**
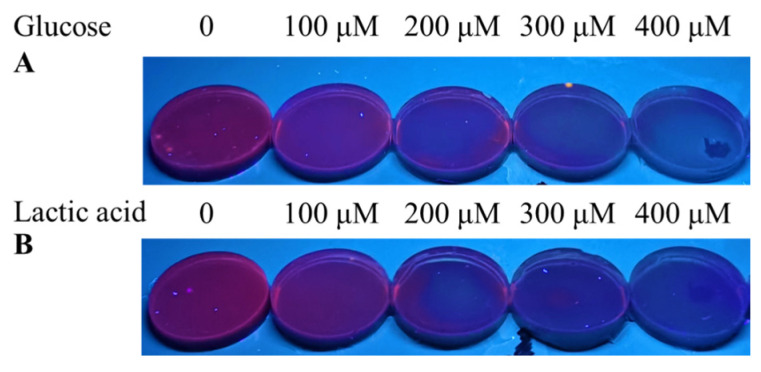
(**A**) The photo of agarose gel discs based on Fe NCs and GluOX immersed in glucose solution with various concentrations for 60 min under UV light. (**B**) The photo of agarose gel discs based on Fe NCs and LacOx immersed in lactic acid solution with various concentrations for 60 min under UV light.

In order to utilize the detection system for glucose or lactic acid conveniently, we tried to make it into an agarose gel disc. As shown in Figure 10A, the Fe NCs solution exhibited bright red fluorescence under UV light. We injected various volumes of Fe NCs solution (1 mg·mL^−1^) directly into the hot agarose solution. After cooling to room temperature naturally, the agarose gel disc based on Fe NCs can be obtained. Figure 10B indicates that the series of agarose gel disc containing various amounts of Fe NCs can exhibit red fluorescence under UV light. Therefore, we chose 400 µL of Fe NCs to prepare the agarose gel disc. The prepared agarose gel discs containing Fe NCs were placed on the plastic plate, and 200 µL of different concentrations of H_2_O_2_ solution (0–40 µM) were injected into these agarose gel discs, respectively. After 30 min of incubation, the color changes of the agarose gel discs were observed by a dark-box ultraviolet analyzer upon 365 nm irradiation. Figure 10C offered the photo of their color changes, and it can be seen that the agarose gel disc changed from red to violet along with the increase in H_2_O_2_ concentrations from 0 to 40 μM. The above results demonstrated feasibility of the agarose gel based on Fe NCs for detecting H_2_O_2_. We further tested the performance of the agarose gel based on Fe NCs and GluOx/LacOx for the visual detection of glucose and lactic acid. As described in Section 2.6 and Section 2.7, we, respectively, prepared agarose gel discs combining Fe NCs and GluOx or LacOx. Figure 11A showed that the agarose gel discs comprising Fe NCs and GluOx can change from red to violet after incubating them with the increasing glucose concentration from 0 to 400 μM under UV light. Similarly, the agarose gel discs comprising Fe NCs and LacOx also changed from red to violet after incubating with the increasing lactic acid concentration from 0 to 400 μM. The above results demonstrated the Fe NCs and the GluOx or LacOx system can be successfully made into an agarose gel disc for convenient detection of glucose or lactic acid.

In order to assess the practicability of this method in real samples, the Fe NCs–GluOx and Fe NCs–LacOx systems were used to detect glucose and lactic acid levels in fetal calf serum samples and milk samples. Fetal calf serum was diluted 20 times, and additional glucose or lactic acid was spiked in the samples. As shown in Table 1 and Table 2, the detection performance for glucose and lactic acid based on this method utilizing the standard addition method was tested. In Table 1, the recoveries of glucose ranged from 95 to 109% with a relative standard deviation (RSD) not exceeding 4.6%. In Table 2, the recoveries of lactic acid were within the range from 90 to 106% with RSD not exceeding 4.2%. Similarly, milk was diluted 20 times, and additional glucose or lactic acid was spiked in the samples. As shown in Table 3 and Table 4, the detection performance for glucose and lactic acid based on this method utilizing the standard addition method was tested. In Table 3, the recoveries of glucose ranged from 92 to 102% with a relative standard deviation (RSD) not exceeding 4.7%. In Table 4, the recoveries of lactic acid were within the range from 91 to 104%, with RSD not exceeding 4.1%. The above results demonstrate the potential practical application of this method.

## 4. Conclusions

We demonstrated that the prepared Fe NCs exhibited excellent peroxide-like activity, and their fluorescence intensity could be significantly quenched by H_2_O_2_. Based on these characteristics, we developed a mixed system composed of Fe NCs and GluOx for detecting glucose in the range of 0–200 μM. Similarly, we also established a detection system composed of Fe NCs and LacOx that was successfully used for detecting lactic acid in the range of 0–100 μM. The proposed methods have satisfactory detection performance in real samples. Moreover, Fe NCs and GluOx were processed as agarose gel discs, which can be used conveniently for visual detection of glucose under UV light. Similarly, Fe NCs and LacOx can also be processed as agarose gel discs, which were used conveniently for the visual detection of lactic acid. The prepared agarose gel discs offered a simple detection mode for glucose and lactic acid, which could be used for the quick judgment of glucose or lactic acid concentration range on the basis of the color changes after the addition of tested samples under UV light.

## Figures and Tables

**Table 1:** Determinations of glucose in fetal bovine serum samples.

Samples	Initial (µM)	Added (µM)	Found (µM)	Recovery (%)	RSD (*n* = 3, %)
1	12.6	5	18.1	110	4.3
2	12.6	10	22.1	95	3.9
3	12.6	20	34.3	109	4.6

**Table 2:** Determinations of lactic acid in fetal bovine serum samples.

Samples	Initial (µM)	Added (µM)	Found (µM)	Recovery (%)	RSD (*n* = 3, %)
1	78.6	5	84.0	108	3.6
2	78.6	10	87.6	90	4.2
3	78.6	20	99.7	106	3.7

**Table 3:** Determinations of glucose in milk samples.

Samples	Initial (µM)	Added (µM)	Found (µM)	Recovery (%)	RSD (*n* = 3, %)
1	15.2	5	20.3	102	3.2
2	15.2	10	24.9	97	4.7
3	15.2	20	33.6	92	2.8

**Table 4:** Determinations of lactic acid in milk samples.

Samples	Initial (µM)	Added (µM)	Found (µM)	Recovery (%)	RSD (*n* = 3, %)
1	69.1	5	74.3	104	3.8
2	69.1	10	78.2	91	4.1
3	69.1	20	88.3	96	3.3

## Data Availability

Data are contained within the article.
